# Tracheotomy in Childhood

**Published:** 1856-04

**Authors:** M. Guersant


### *Tracheotomy in Childhood.* By M. Guersant.

(L'Union Medicale, Jan. 16, 1855.)

In this paper M. Guersant calls attention to the greater frequency with which he has been called upon to perform this operation at the Hopital des Enfans Malades. Thus, in 1850, there were 10 operations; in 1851, 25; in 1852, 30; and in 1853, 60. M. Guersant concludes from these facts that croup is on the increase in Paris; a conclusion doubted by M. Denonvilliers, who ascribes these increasing numbers to the greater confidence of the public in the resources of surgery in such cases. As regards the success of the operation, M. Guersant remarks that out of 161 cases in which it was performed there were 36 cures; and it must be borne in mind that it was only performed as a last resource when asphyxia was considered to be imminent. The rest of the treatment was much the same as is usual in this country. M. Guersant also frequently employed cauterization of the larynx, but considers that this treatment does not deserve the credit it has attained.

M. Archambault directs attention to a peculiar phenomenon which frequently attends on recovery in those cases of croup in which tracheotomy is performed. As soon as the glottis is free from false membranes, it is observed that the infant, in swallowing, is extremely apt to be distressed by the passage of fluid matters into the larynx. This difficulty of deglutition has occurred in the practice both of M. Trousseau and of M. Guersant. The former attempts to remove it by giving only solid or semi-solid food; the latter proposes in extreme cases to pass the œsophagus tube. M. Archambault recommends a simpler method. He removes the canula, and applies the thumb very firmly over the opening. When the child has made several respirations, and these have become regular and natural, he allows it to drink, after directing its attention to the necessity of caution. In this way drink can be taken. M. Archambault considers that this difficulty of deglutition depends on an impaired sensibility of the glottis, and a want of harmony between the act of deglutition and respiration, owing to the artificial manner in which the latter has been effected through the canula. He has not witnessed this symptom except when the respiration was notably accelerated.—*Ranking's Abstract.*

